# Impaired function of dendritic cells within the tumor microenvironment

**DOI:** 10.3389/fimmu.2023.1213629

**Published:** 2023-06-27

**Authors:** Zhihua Xiao, Ruiqi Wang, Xuyan Wang, Haikui Yang, Jiamei Dong, Xin He, Yang Yang, Jiahao Guo, Jiawen Cui, Zhiling Zhou

**Affiliations:** ^1^ Department of Pharmacy, Zhuhai People’s Hospital (Zhuhai Hospital Affiliated with Jinan University), Zhuhai, China; ^2^ College of Pharmacy, Jinan University, Guangzhou, China

**Keywords:** dendritic cell, tumor microenvironment, immune tolerance, immunosuppressive populations, DC-based vaccine

## Abstract

Dendritic cells (DCs), a class of professional antigen-presenting cells, are considered key factors in the initiation and maintenance of anti-tumor immunity due to their powerful ability to present antigen and stimulate T-cell responses. The important role of DCs in controlling tumor growth and mediating potent anti-tumor immunity has been demonstrated in various cancer models. Accordingly, the infiltration of stimulatory DCs positively correlates with the prognosis and response to immunotherapy in a variety of solid tumors. However, accumulating evidence indicates that DCs exhibit a significantly dysfunctional state, ultimately leading to an impaired anti-tumor immune response due to the effects of the immunosuppressive tumor microenvironment (TME). Currently, numerous preclinical and clinical studies are exploring immunotherapeutic strategies to better control tumors by restoring or enhancing the activity of DCs in tumors, such as the popular DC-based vaccines. In this review, an overview of the role of DCs in controlling tumor progression is provided, followed by a summary of the current advances in understanding the mechanisms by which the TME affects the normal function of DCs, and concluding with a brief discussion of current strategies for DC-based tumor immunotherapy.

## 1 Introduction

Dendritic cells (DCs), first discovered by Steinman and Cohn in 1973 (1), serve as a bridge between innate and adaptive immunity in the host immune response. Based on differences in the expression of cell surface markers, DCs can be divided into two main subgroups: conventional DCs (cDCs) and plasmacytoid DCs (pDCs), each with a unique function in immune activity (2). cDCs have powerful antigen capture and presentation capacities and are one of the mainstays of T-cell activation in the body. In contrast, pDCs can present antigens to T-cells, although not as efficiently as cDCs. The main characteristic of pDCs is that they can direct the immune response by secreting high levels of type I interferons (IFN-I) (3, 4). Furthermore, DCs have been extensively studied, and their central role in initiating and maintaining anti-tumor immune responses to hinder tumor progression has been well established. However, the tumor microenvironment (TME) shows characteristics that are different from those of normal tissues, including the infiltration of a large population of immunosuppressive cells and a unique environment of hypoxia and lactate accumulation (5–7), rendering DCs incompetent by impairing their maturation, limiting their antigen capture, and downregulating the expression of costimulatory molecules in a variety of ways (8, 9). In this review, the essential role of DCs in tumor immunosurveillance is discussed, and the mechanisms by which the TME affects the function of DCs in tumors are summarized. Finally, we evaluated the improvement in DC-based tumor immunotherapy strategies, particularly DC-based vaccines.

## 2 The role of dendritic cells in tumor immunosurveillance

**Figure 1:**
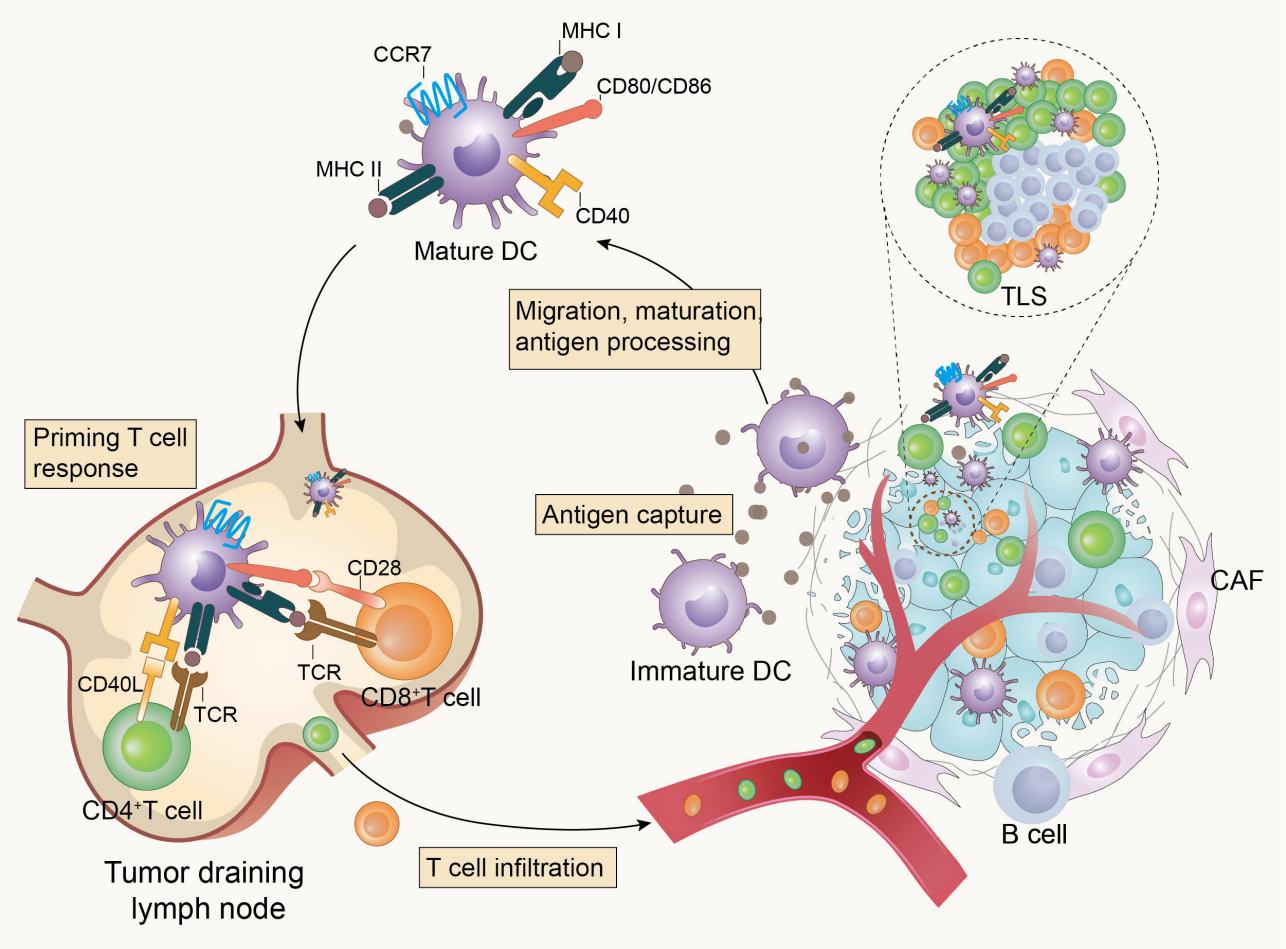
Dendritic cells initiate anti-tumor immunity. Tumor-infiltrating dendritic cells recognize and capture tumor-associated antigens, then become mature and homing to tumor-draining lymph nodes (TDLNs) or tertiary lymphoid structures (TLS) to activate T-cells and initiate anti-tumor immunity in response to the presence of tumors.

Effective anti-tumor immune responses involve a series of stepwise events. Chen et al. summarized the complex anti-tumor immune process as the “Cancer-Immunity Cycle” (reviewed in (10)), which provides an important framework for understanding the overall picture of the anti-tumor immune process. Furthermore, DCs are pivotal in the overall anti-tumor immune response due to their key role in T cell activation and immune response initiation (**Figure 1**). Briefly, immature DCs that infiltrate the tumor tissue recognize and phagocytose apoptotic or necrotic tumor cells and thus tumor cell antigens. They subsequently enter an activation/maturation process triggered by an intrinsic program and migrate from the tumor tissue *via* the lymphatic vessels or blood circulation to tumor-draining lymph nodes (TDLNs). During migration, DCs mature and acquire new characteristics, including the upregulation of CC-chemokine receptor 7 (CCR7) for improved motility, the upregulation of major histocompatibility complex (MHC) class I and class II molecules for antigen presentation, upregulation of costimulatory molecules such as CD80, CD86, and CD40, and increased cytokine secretion for enhanced T-cell stimulation. Mature DCs load endo-processed antigenic peptides onto MHC class I or MHC class II molecules for presentation to naïve T-cells, and at the same time, the costimulatory molecules interact with the ligands on T cells, which synergistically stimulate the activation and differentiation of T-cells in TDLNs (3, 11–17). Tertiary lymphoid structures (TLS), which are crucial in the anti-tumor immune response, may also be the destination for the migration of mature DCs (18, 19). The TLS may represent a privileged site for the local presentation of neighboring tumor antigens to T-cells by DCs and the activation, proliferation, and differentiation of T-cells (19, 20). This is also supported by a single-cell analysis of human non-small cell lung cancer lesions, which showed that mature DCs enriched in immunoregulatory molecules (mregDCs) accumulated in the TLS in close proximity to T-cells (21). MregDCs are a new cluster of DCs identified by Maier et al. in human and mouse non-small cell cancers and are characterized by the expression of both maturation markers and regulatory molecules (22). MregDCs have also been described in various human cancers, including hepatocellular carcinoma (23), breast cancer (24), colon cancer (25), and gastric cancer (26). Li et al. summarized the basic characteristics of mregDCs and suggested that lysosomal-associated membrane protein 3 (LAMP3) may be a fundamental recognition marker for them (27). Ginhoux et al. proposed that mregDCs can refer to a distinct molecular state induced in cDC1s, cDC2s, and potentially inflammatory DC3s upon sensing or capturing cell-associated materials that have a distinct ability to interact with antigen-specific T-cells (28). Analysis of tumors and metastatic lymph nodes from patients with head and neck lymphoma revealed that mregDCs may contribute to the prognosis by balancing regulatory and effector T-cells (29).

It is well established that DCs play a key role in stimulating cytotoxic T-cells and driving immune responses against cancer and that the levels of intratumoral stimulatory DCs in human tumors are associated with increased overall survival (30–32). Hegde et al. suggested that different scales of infiltration of cDCs would induce different levels of T-cell responses and that increased infiltration and activation of cDCs enhanced the activity of CD8^+^ T and T_H_1 cells in a pancreatic cancer mouse model (33). In addition, further evidence for the role of DCs in controlling tumor development is derived from the fact that the absence and dysfunction of DCs in tumor-bearing mouse models lead to poorer outcomes and insensitivity to anti-tumor treatment. Batf3-deficient mice (*Batf3^-/-^*) lack cross-presenting DCs and fail to trigger cytotoxic T lymphocyte-mediated immune responses to tumor-associated antigens (34–36), and Mittal et al. observed increased tumor metastasis and poorer survival in *Batf3^-/-^* mouse models of breast cancer and melanoma than in wild-type mice (37). Furthermore, it has been observed in several *Batf3^-/-^* mouse models that activated DCs are required to promote the anti-tumor efficacy of immunostimulatory antibodies, such as anti-PD-1, anti-PD-L1, and anti-CD137, and deficiencies in DCs limit the efficacy (35, 38). This suggests that the functional status of DCs is closely related to the efficacy of tumor immunotherapy. pDCs have a weak antigen-presenting capacity but can participate in the tumor immune response in other ways, such as by secreting IFN-I (39) and cross-priming naïve CD8^+^ T-cells by transferring antigens to cDCs *via* exosomes (40). However, the function of pDCs in TME remains controversial. In patients with colon cancer, an increased density of infiltrating pDCs was significantly correlated with increased progression-free and overall survival (41). In addition, a naturally occurring pDCs subset expressing high levels of OX40 with a unique immunostimulatory phenotype was identified in the TME of patients with head and neck squamous cell carcinoma, which, when synergized with cDCs, generated potent tumor antigen-specific CD8^+^ T-cell responses (42). However, as reported by Sisirak and partners, tumor-infiltrating pDCs in patients with breast and ovarian cancer are associated with poor outcomes (43, 44), and this may be linked to tumor cell-derived cytokines such as TGF-β and TNF-α, which limit the ability of pDCs to produce IFN-I and induce them to be tolerogenic (45, 46). The specific microenvironmental context and functional status of pDCs appear to determine their effects on cancer immunity and patient outcomes.

Overall, the evidence indicates that DCs, although representing a relatively rare subset of immune cells, are an essential part of anti-tumor immunogenesis. Moreover, when functionally activated, they are associated with stalled tumor progression and improved therapeutic responsiveness. However, the prognostic role of DCs in patients with cancer cannot be generalized and is largely dependent on the density, maturation, and activity of DCs. In general, tumor infiltration by activated, well-functioning DCs tends to predict a better prognosis, whereas DCs with impaired functional status in the TME may have the opposite effect on tumor progression (47–50). The TME causes the loss of antigen presentation and T-cell stimulatory capacity by inhibiting the maturation and migration of DCs, altering their ability to secrete cytokines. This can even induce tolerogenic or immunosuppressive DCs, allowing the tumor to escape surveillance and extermination by the immune system.

## 3 Immunosuppressive effects of the TME on dendritic cells

**Figure 2:**
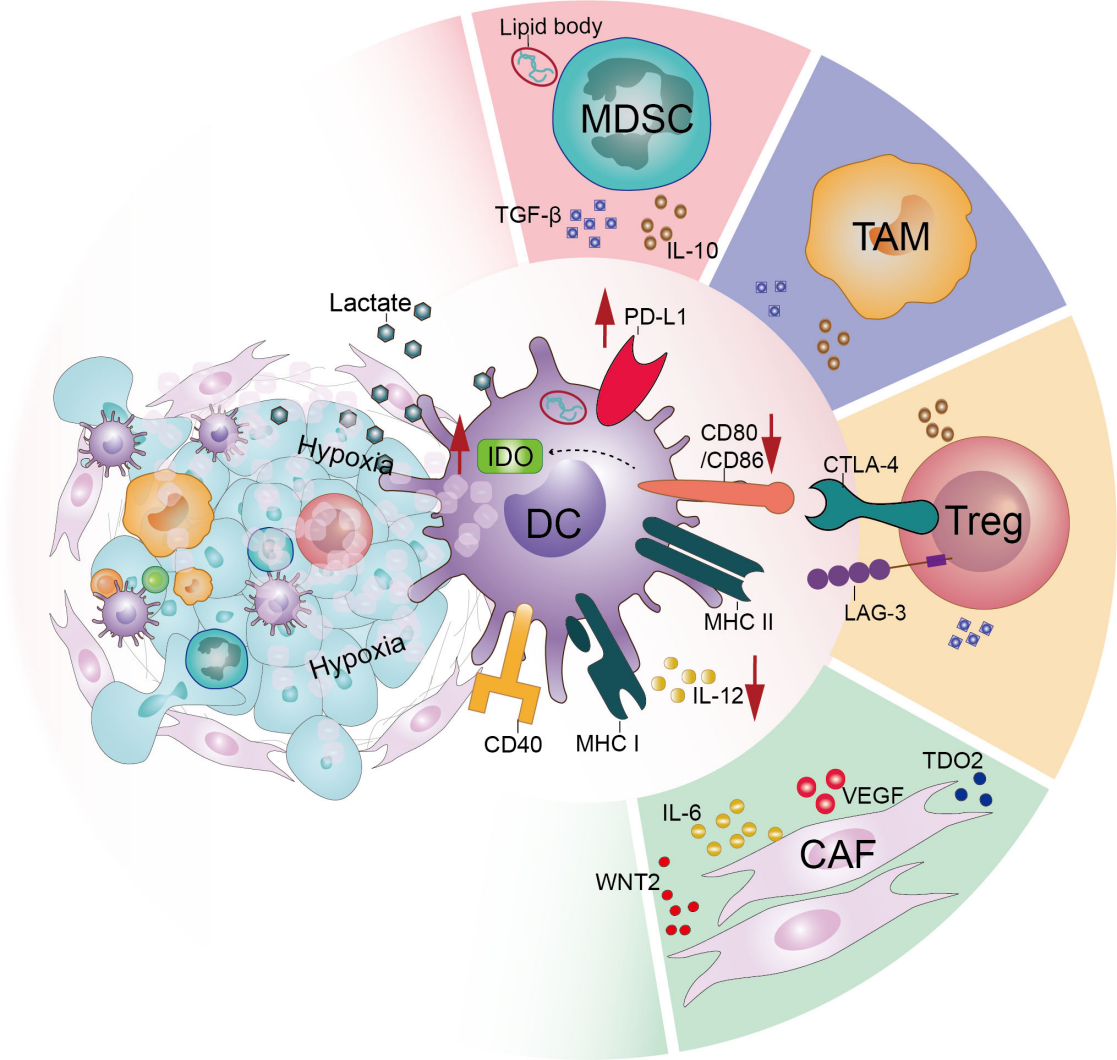
Tumor microenvironment acts on dendritic cells and downregulates their function. In the tumor microenvironment, various factors interact directly or indirectly with dendritic cells to dysfunction them. These include the large number of immunosuppressive populations such as regulatory T-cells (Tregs), tumor-associated macrophages (TAMs) and myeloid-derived suppressor cells (MDSCs) infiltrating the tumor microenvironment. In addition, the effects of stromal cells such as cancer-associated fibroblasts (CAFs) and the particular hypoxic and acidic microenvironment of the tumor microenvironment cannot be ignored.

The conditions for tumor development, metastasis, and invasion are provided by the TME, a complex and dynamically evolving system composed of numerous components, including tumor cells, immune cells, the extracellular matrix, and soluble cytokines. Accumulating evidence indicates that immunosuppressive populations and stromal cells, as well as the unique metabolic environment of the TME, negatively regulate the maturation, migration, and effector functions of DCs (**Figure 2**).

### 3.1 Inhibition of dendritic cells by immunosuppressive populations

One of the most prominent features of the TME is the progressive accumulation of tumor-associated immunosuppressive cell populations, such as regulatory T-cells (Tregs), myeloid-derived suppressor cells (MDSCs), and tumor-associated macrophages (TAMs) (51, 52).

Aberrant chemokine alterations in the TME are important in the tumor recruitment of immunosuppressive cells (53). Tumor cells can induce the migration of Tregs to the TME by upregulating the expression of several chemokines, including the C-C motif chemokine ligand (CCL) 17/22 (54), CCL20 (55), and CCL28 (56, 57). Moreover, the ability of Tregs to use free fatty acids and lactate allows them to survive and maintain their suppressive identity, particularly in a harsh nutrient TME (58, 59). Tregs are a major suppressor group that induce DCs dysfunction and limit tumor immunogenesis (60). One important mechanism by which Tregs cause DCs dysfunction is through cytotoxic T-lymphocyte-associated antigen 4 (CTLA-4). Tregs expressing CTLA-4 compete with CD28 on conventional T-cells for the co-stimulatory molecules CD80 and CD86 on the surface of DCs, with CTLA-4 having a greater affinity and avidity than CD28 (61). In addition, Tregs are able to downregulate CD80/CD86 molecules expressed by DCs in a CTLA-4-dependent manner (62–65), and depletion of CD80/86 in mice was also found to cause upregulation of PD-L1 in DCs (66), resulting in multiple inhibitory effects on DC-mediated T-cell immune responses. Furthermore, the interaction of CTLA-4 with CD80/CD86 induces the production of indoleamine-2,3-dioxygenase (IDO) in DCs, which can induce tryptophan catabolism to pro-apoptotic metabolites, leading to the suppression of effector T-cell activation (67–69). In addition to CTLA-4, lymphocyte activation gene-3 (LAG-3), an immune checkpoint molecule that has recently received considerable attention, is constitutively expressed on Tregs and can limit the T cell stimulatory capacity of DCs by interacting with MHC class II molecules (70, 71). A number of other interactions, including the secretion of inhibitory cytokines such as IL-10 and TGF-β (72), delivery of miRNAs to DCs by secreted extracellular vesicles, thereby inducing a tolerogenic phenotype in DCs (73), expression of CD27 molecules that interfere with CD70/CD27 stimulatory signaling between DCs and effector T-cells (74), and direct induction of death through mutual contact with DCs (75), are also important means for Tregs to impede the onset of DCs-mediated tumor immunity. Consistently, enhanced anti-tumor immune responses induced by DCs have been observed after reducing the infiltration of tumor-associated Tregs and the secretion of their immunosuppressive molecules in various tumor-bearing mouse models (67, 76–78). Thus, Tregs appear to be an important cell subpopulation in the TME that acts directly on DCs and mediates their dysfunction, so the depletion of Tregs may be beneficial for DCs to mediate anti-tumor immunity.

MDSCs are a heterogeneous population of immature myeloid cells with immunosuppressive properties. Under the stimulation of the pathological conditions of cancer, the maturation and differentiation of bone marrow-derived progenitor cells are blocked, resulting in the accumulation of immunosuppressive MDSCs. MDSCs are recruited to the TME *via* multiple chemokine signals such as CCL2, CCL5, CCL26, C-X-C motif chemokine ligand (CXCL) 8, CXCL12, and other mediators such as granulocyte-macrophage colony-stimulating factor (GM-CSF), IL-6, or prostaglandin E2 (PGE2) that participate in expanding MDSCs (79). Previous studies have shown that activated MDSCs impede anti-tumor immunity and promote tumor progression through a series of actions, and that DCs are negatively affected (80). Hu et al. observed that upregulated MDSCs were associated with higher IL-10 expression, lower IL-12 production by DCs, and lower T-cell stimulatory activity in mice with hepatocellular carcinoma (81). Furthermore, it has been reported that tumor-associated DCs accumulate large amounts of lipid bodies (LB) containing oxidized lipids, impeding cross-presentation in DCs by covalently binding to heat shock protein 70 and preventing the translocation of peptide-MHC I complexes (pMHC) to the cell surface (82–84). Ugolini et al. found that in tumor-bearing mice, polymorphonuclear (PMN)-MDSCs are able to transfer lipid bodies to DCs, causing them to exhibit impaired antigen cross-presentation. Consistently, in MDSCs depleted or myeloperoxidase (MPO, a key enzyme for the production of oxidized lipids in MDSCs) deficient mice, DCs showed improved activity for tumor antigens cross-presentation (85). Thus, it appears that the abnormally large accumulation of lipids and impaired antigen cross-presentation in DCs are at least partially related to MDSCs and that selective depletion of MDSCs may be a potential option for restoring the function of DCs in tumor conditions.

In many solid tumor types, TAMs are among the most abundant populations of tumor-infiltrating immune cells in the TME (86). TAMs may localize to the TME either by traveling *via* chemotactic gradients regulated by factors such as CCL2, IL-1β, and macrophage colony-stimulating factor 1 (CSF1), differentiating from monocytes in the TME or by repolarization of tissue-resident macrophages (87). In addition, TAMs in the TME are more inclined to polarize into an anti-inflammatory phenotype due to the influence of cytokines such as PGE2 (88–90). TAMs are involved in multiple aspects of immunosuppression, and a high infiltration of TAMs into solid tumors is usually associated with a poor prognosis (86, 91–93). Unlike Tregs, which interact directly with DCs, TAMs mediate the recruitment of other immunosuppressive cells and secrete inhibitory cytokines that influence the maturation and function of DCs (94). Ruffell et al. described that in the TME of breast cancer mice, TAMs inhibit the production of IL-12 by DCs through the secretion of IL-10, attenuating the cytotoxic CD8^+^ T-cell response (95). Several preclinical studies have also suggested that TAM depletion in the TME can reshape the link between DCs and T-cells. For example, in a study based on a murine model of lung cancer, after targeting macrophages with a CSF1R inhibitor (CSF1Ri), the authors observed increased crosstalk between immunostimulatory populations, including DCs, NK cells, and T-cells, and increased levels of IL-12 expressed by DCs and T-cells, respectively (96). TAMs were consistently targeted by CSF1Ri (PLX3397) in a mouse model of mesothelioma. When combined with a DC-based vaccine, a robust and durable anti-tumor immune response was observed (97).

### 3.2 The function of dendritic cells is limited by stromal cells

Tumor progression and immune tolerance cannot be achieved without the involvement of tumor stromal components (98). Cancer-associated fibroblasts (CAFs), a complex and heterogeneous cell population, are the most abundant components of a tumor stroma. Tissue-resident fibroblasts are the major sources of CAFs (99), which can be activated by stimulation of various factors of TME such as TGF-β, TNF, fibroblast growth factor, and platelet-derived growth factor (100, 101). Additionally, mesenchymal stem cells, epithelial cells, and endothelial cells adjacent to cancer cells and fibroblasts recruited from the bone marrow are potential sources of CAFs (102, 103). The interaction of CAFs with immune cells has been identified as a key contributor to tumor progression. Several recent studies have revealed that CAFs can drive the immune escape of tumor cells by impeding the maturation, migration, and antigen presentation of DCs. Berzaghi et al. reported that the co-incubation of CAFs obtained from surgically resected fresh tumor tissue from lung cancer patients with mature DCs results in impaired migration and antigen uptake (104). In another study, it was proposed that human lung cancer cell-stimulated CAFs impair the differentiation and function of DCs by upregulating tryptophan-2,3-dioxygenase (TDO2) (105). Cheng et al. found that *in vitro* hepatocellular carcinoma patient-derived CAFs can recruit normal DCs and mediate STAT3 pathway activation by expressing IL-6, inducing their transformation into regulatory DCs (106). Furthermore, CAFs secrete abundant active factors such as vascular endothelial growth factor (VEGF), which promote angiogenesis while mediating damage to the migratory and T-cell stimulatory capacities of DCs (107, 108). Excellent work was reported by Huang et al., who found that CAF-secreted WNT2 was involved in the differentiation and immunostimulatory activity of DCs *in vitro*, and accordingly, anti-WNT2 was observed to increase the level of intratumoral activated DCs and significantly improve the anti-tumor responses of DC-mediated antigen-specific CD8^+^ T cells in murine tumor models (109). This suggests that in the TME, both stromal cells and immunosuppressive cells influence anti-tumor immunity. Therefore, for effective tumor therapy, it is essential to consider targeting stromal cells.

### 3.3 Environmental factors that regulate dendritic cell function in the TME

Compared with normal tissues, the TME exhibits a significantly hypoxic and acidic environment and is an important mediator of tumor progression.

Hypoxia is a central player in shaping the immune context of the TME, which results from an imbalance between increased oxygen consumption and inadequate oxygen supply owing to the rapid proliferation of tumor cells (110). Many physiological functions of DCs, including migration and maturation, are regulated by hypoxia. Hypoxic immature DCs exhibit upregulated motility/migration ability (111), while their antigen uptake ability is seemingly downregulated (112, 113). Consistently, Suthen et al. observed significant enrichment of Tregs and cDC2 in hypoxic regions of tumor samples from patients with HCC, as well as lower CD8^+^ T-cells, and found a significant downregulation of HLA-DR expression by cDC2 under hypoxic conditions, which may be related to the increased intercontact between Tregs and cDC2 during hypoxia (114). Besides, it is well known that hypoxia-inducible factor-1alpha (HIF-1α) plays a key role in the cellular response to hypoxia (115, 116), yet the effects of HIF-1α on DCs appear to be controversial. On the one hand, several scholars have demonstrated that the increase in HIF-1α in DCs under hypoxia is accompanied by an increase in the expression of HIF-1α target genes, including those involved in glycolysis, and that the increase in glycolysis will promote the maturation and migration of DCs (117–119). On the other hand, however, it has been proposed that constitutive expression of HIF-1α impairs the immunostimulatory capacity of DCs *in vivo* by inducing DCs to upregulate the expression of immunosuppressive mediators such as IL-10, iNOS, and VEGF (120, 121). Additionally, prolonged exposure to hypoxia induces cell death in DCs, which can be prevented by HIF-1α inhibition, suggesting that HIF-1α may be involved in this process (122). It was observed in human glioma cells that hypoxia induces PD-L1 upregulation in an HIF-1α-dependent manner, and it was further found in a murine glioma model that the combination of HIF-1α inhibitor and anti-PD-L1 antibody can improve the activation of DCs and CD8^+^ T-cells (123). Notably, hypoxic conditions recruit more immunosuppressive Tregs (56, 114) and TAMs (124), thereby indirectly curbing the function of DCs. Overall, hypoxia appears to facilitate the migration and maturation of DCs and compromise their normal functions. The exact changes in the behavior of DCs under hypoxic conditions need to be further elucidated.

Tumor cells exhibit altered metabolism, preferentially converting glucose to lactate through glycolysis even under oxygen-rich conditions. This results in a large accumulation of lactate and increases the acidity of the TME (125–127). Numerous studies have shown that lactate accumulation in the TME adversely affects the DC function. For example, tumor-derived lactate restricts the presentation of tumor-specific antigens by DCs to other immune cells (128). Lactate is also involved in regulating the phenotype of DCs, resulting in increased production of anti-inflammatory cytokines and decreased production of pro-inflammatory cytokines (129, 130). In patients with melanoma, the function of pDCs is impaired by lactic acidosis (131), with the same phenomenon observed in patients with breast cancer and murine models (132). Some researchers have suggested that in mice, the migratory capacity of DCs is significantly diminished in acidic environments and does not recover after removal of the acidic microenvironment, suggesting that extracellular acidosis may cause irreversible DCs dysfunction (133). In addition, exposure of mesothelioma cells to acidosis promotes the secretion of TGF-β2, which in turn leads to the accumulation of lipid droplets in DCs, resulting in a reduction in DC migratory capacity (134). These findings support the view that an acidic environment is not conducive to the proper functioning of DCs. However, Geffner et al. argued that extracellular acidosis stimulates antigen capture, promotes the expression of MHC class II molecules CD86 and CD40, and induces the maturation and secretion of IL-12 in mouse (135) and human DCs (136). Notably, the maintenance of an acidic environment and the accumulation of lactate in the TME complement each other. In tumors, an acidic environment can promote the accumulation of lactate and thus impair the function of DCs.

In general, owing to the combination of many factors in the TME, DCs are significantly dysfunctional. An accurate understanding of the role of each component in DC dysfunction will help to better understand the tumor state and to accurately explore ways to restore the activity of DCs. However, the TME is a complex and interconnected whole, and ultimately, all factors need to be linked for a systematic and comprehensive understanding of the causes and processes of the dysfunction of DCs.

## 4 Dendritic cell-based strategies for cancer immunotherapy

As the key activators of the immune response, the immune activation potential of DCs can be used to induce anti-tumor responses in patients with cancer, which is a promising development. Primary strategies based on DCs include the creation of immunoenhancers that promote the generation and activation of DCs, or the preparation of autologous DC-based vaccines for patient administration. Flt3L, GM-CSF, and Toll-like receptor (TLR) ligands are common immunoenhancers. The development and maintenance of DCs depend on the Flt/Flt3L axis (137), and attempts have been made in clinical studies to enhance the immune response induced by tumor vaccines by administering Flt3L (NCT02129075) (138). GM-CSF stimulates the differentiation, activation, and migration of DCs (139, 140), and consistently, administration of the CpG ODN/GM-CSF combination in melanoma patients results in enhanced mutation of all identifiable DC subpopulations and the recruitment of T-cell-stimulating and cross-presenting DCs to support protective melanoma immunity (141). When combined with TLRs in DCs, TLR ligands can activate signal transduction pathways and induce the expression of genes involved in the maturation of DCs (142). Therefore, some immunostimulatory ligands for TLRs, such as poly(I:C), are often used as immunoadjuvants in DC-based therapies and have shown promising results (143, 144). DEC205, also known as CD205 or LY75, is an endocytic receptor expressed at high levels by CD8^+^ DCs and is involved in antigen uptake and cross-presentation (145). The fusion of tumor antigens with targeted antibodies against DEC205 to enhance DC-induced immune responses has been well studied and explored in clinical trials (138, 146). Recently, a pioneering study provided new insights into the application of DEC205 as a therapeutic target. Martinek et al. analyzed the transcriptome of T-cells and macrophages *in situ* in melanoma patient samples using immunofluorescence-guided laser capture microdissection and observed that stromal macrophages contained a gene expression signature linked to antigen capture and presentation (*CD14^+^LY75^+^*). This can distinguish patients with significantly better long-term survival and includes a gene module of monocyte-derived DCs (147). This study provides valuable insights into the reprogramming of stromal macrophages to upregulate gene features related to antigen capture and presentation to acquire DCs function and could be a potential option for cancer therapy.

DC-based therapeutic cancer vaccines are a popular strategy for stimulating an effective tumor immune response as they return autologous activated DCs loaded with tumor-associated antigens to patients (148). In April 2010, the FDA approved the marketing of the first DCs vaccine, sipuleucel-T, for the treatment of prostate cancer (149). Furthermore, in the NCCN Clinical Practice Guidelines in Oncology (NCCN Guidelines^®^): Prostate Cancer (version 1.2023), sipuleucel-T is recommended for the treatment of metastatic castration-resistant prostate cancer (CRPC) and is a category 1 option for certain patients who have not received previous treatment with docetaxel or novel hormone therapy. Sipuleucel-T is also an option for patients with metastatic CRPC who have received prior treatment with docetaxel or a novel hormone therapy, but not for patients who have already received both (150). In recent years, DC-based vaccines have undergone extensive clinical trials for the treatment of various cancers, including liver cancer (151), melanoma (152), lung cancer (153), ovarian cancer (154), and pancreatic cancer (155). Although the safety of DC-based vaccines has been proven over the past few decades, their clinical efficacy requires improvement. Consequently, DC-based vaccines are undergoing a great deal of technical innovation, including the selection of DC subpopulations, methods of induction maturation, and choice of loading antigens (148, 156), with the aim of exploiting the anti-tumor potential of DCs more effectively.

The key to cancer immunotherapy is the manipulation of the immune system to achieve cancer control and the desired treatment. The efficacy of immune checkpoint inhibitors, which have shown some success, depends largely on the present baseline immune response, and DC-based vaccines are highly effective at rescuing the baseline anti-tumor immune response. Therefore, there has been considerable interest in combining DC-based vaccines with immune checkpoint inhibitors (ICIs), and several such studies have been conducted in recent years (**Table 1**). Recently, Guo et al. reported a case of a patient with metastatic gastric cancer whose tumor progressed after the first two months of receiving personalized neoantigen-loaded monocyte-derived dendritic cell (Neo-MoDC) vaccine alone, despite the observed T-cell response against the tumor neoantigen and the fact that upregulated PD-1 levels in T-cells were observed after Neo-MoDC vaccine administration. Subsequently, the patient received a combination treatment of the Neo-MoDC vaccine and nivolumab; promisingly, the combination triggered a stronger immune response and mediated complete regression of all tumors for over 25 months (157). Furthermore, anti-PD-1/PD-L1 antibodies in combination with DC-based vaccines have been extensively explored in a variety of murine tumor models (158–164) and, without exception, combination treatment has shown superior efficacy compared to monotherapy, with stronger anti-tumor-specific T-cell responses and lower immunosuppressive cell infiltration. Additionally, the combination of anti-CTLA-4 and DC-based vaccines could lead to more effective cancer treatments. For example, in a clinical trial (NCT01302496), researchers enrolled 39 patients with pretreated advanced melanoma who received a DC-based mRNA vaccination plus ipilimumab. The results showed that a strong tumor-associated antigen-specific immune response was observed in patients treated with the combination of a DC-based vaccine and ipilimumab, with an encouraging 6-month overall response rate of 38%. Subsequent long-term follow-up after more than 5 years indicates that 7/39 patients, who all achieved a complete response, were still disease-free (165). Similarly, in the exploration of multiple preclinical experimental models of pancreatic cancer (166), breast cancer (167), colorectal cancer (168), and melanoma (169), the silencing of CTLA-4 can induce a more effective anti-tumor immune response together with DC-based vaccines by reducing the infiltration of immunosuppressive cells and increasing the Teff/Treg ratio. In summary, combining DC-based vaccines with immune checkpoint inhibitors is a promising option for treating tumors.

**Table 1:** Active clinical trials combining DC-based vaccine with immune checkpoint inhibitors (ICIs) therapy (clinicaltrials.gov, April 28, 2023).

Intervention		Tumor	Phase	*N*	Trial identifier	Status
ICIs	DC-based vaccine used					
Pembrolizumab	Anti-HER2/HER3 DC vaccine	Breast cancer	II	23	NCT04348747	Recruiting
	CCL21-gene modified autologous DC vaccine	Non-small cell lung cancer	I	24	NCT03546361	Recruiting
	Autologous DC loaded with autologous tumor homogenate	Mesothelioma	I	18	NCT03546426	Recruiting
	Autologous tumor lysate-pulsed DC vaccine	Glioblastoma	I	40	NCT04201873	Recruiting
	Intra-tumor injection of autologous DC	Non-Hodgkin lymphoma	I/II	11	NCT03035331	Active, not recruiting
	Therapeutic autologous DC	Melanoma	I/II	7	NCT03325101	Active, not recruiting
	Autologous DC pulsed with melanoma tumor-specific peptides	Melanoma	I	12	NCT03092453	Active, not recruiting
Nivolumab	Autologous neoantigen pulsed autologous DC vaccine	Hepatocellular carcinoma and liver metastases from colorectal carcinoma	II	60	NCT04912765	Recruiting
Camrelizumab	Glioblastoma stem-like cell antigens- pulsed DC vaccine (GSC-DCV)	Glioblastoma	II	40	NCT04888611	Recruiting
Atezolizumab	Autologous DC vaccine	Small cell lung cancer	I/II	20	NCT04487756	Recruiting
	DC loaded with the mesothelioma-associated tumor antigen WT1	Pleural mesothelioma	I/II	15	NCT05765084	Recruiting
Nivolumab/ Ipilimumab	DC-based p53 Vaccine	Small cell lung cancer	II	14	NCT03406715	Active, not recruiting
	Tumor-lysate loaded autologous dendritic cells	Glioblastoma	I/II	25	NCT03879512	Recruiting
Anti-PD-1 antibody	Autologous EphA2-targeting CAR-DC vaccine loaded with KRAS mutant peptide (KRAS-EphA-2-CAR-DC)	Solid tumors	I	10	NCT05631899	Recruiting
	Autologous EphA2-targeting CAR-DC vaccine loaded with TP53 mutant peptide (TP53-EphA-2-CAR-DC)	Solid tumors or lymphomas	I	10	NCT05631886	Recruiting
Anti-PD-1/PD-L1 antibody	Alpha-type-1 polarized dendritic cell (αDC1) vaccine	Melanoma	II	24	NCT04093323	Recruiting

Combining a personalized DC-based vaccine with chemotherapeutic agents and targeted drugs is also an effective way to improve the efficacy of tumor vaccines, and we have compiled active relevant clinical trials in **Tables 2**, **3**. It is already clear that chemotherapy can enhance the efficacy of DC-based vaccines by enhancing antigen production and eliminating suppressive immune cells. Some specific chemotherapeutic drugs, such as cyclophosphamide (170), have been shown to directly deplete suppressive immune cells in patients with cancer at low doses. A phase I clinical study suggested that cyclophosphamide with a DC-based vaccine treatment downregulated tumor infiltration of immunosuppressed cells and demonstrated excellent anticancer effects (NCT01241682) (171). In glioblastoma, a combination of Temozolomide- and DC-based vaccines has been favored, and recently, the publication of the results of a phase III prospective externally controlled cohort trial has gained widespread attention (NCT00045968). The results show that the median overall survival for patients with newly diagnosed glioblastoma assigned to the DCVax-L cohort (232 patients, 222 of whom received autologous tumor lysate-loaded dendritic cell vaccine “DCVax-L” plus temozolomide) at enrollment was 19.3 months from the time of randomization compared with 16.5 months from randomization for the 1366-patient external control populations. In addition, in patients with recurrent glioblastoma, the combination of DCVax-L with standard treatment showed a survival benefit (172). Currently, chemotherapy remains the primary treatment for most cancers, and the combination of chemotherapy and DC-based vaccines has promising prospects owing to their cooperative effect. Furthermore, the combination of DC-based vaccines and targeted drugs has been explored. In a phase II clinical trial, Storkus et al. proposed that DC-based vaccines targeting tumor blood vessel antigens combined with dasatinib could induce therapeutic immune responses in patients with checkpoint-refractory advanced melanoma (NCT01876212) (173). Trastuzumab can enhance the uptake and cross-presentation of HER-2 derived peptides by DCs to improve the generation of peptide-specific CTLs (174), which provides a theoretical reference for the combination of Trastuzumab with a DC-based vaccine.

**Table 2:** Active clinical trials combining DC-based vaccine with chemotherapy drugs (clinicaltrials.gov, May 28, 2023).

Intervention		Tumor	Phase	*N*	Trial identifier	Status
Chemotherapy drug(s)	DC-based vaccine used					
Temozolomide	Autologous dendritic cells loaded with autologous tumor homogenate in glioblastoma	Glioblastoma	II	28	NCT04523688	Recruiting
	Malignant glioma tumor lysate-pulsed autologous dendritic cell vaccine	Glioblastoma	I	21	NCT01957956	Active, not recruiting
	Autologous Wilms’ tumor 1 (WT1) messenger (m)RNA-loaded dendritic cell (DC) vaccine	Glioblastoma	I/II	20	NCT02649582	Recruiting
	Dendritic and glioma cells fusion vaccine	Glioblastoma	I/II	10	NCT04388033	Recruiting
	Human CMV pp65-LAMP mRNA-pulsed autologous DCs	Glioblastoma	II	80	NCT03688178	Recruiting
	Autologous dendritic cells pulsed with multiple neoantigen peptides	Glioblastoma	I	10	NCT04968366	Recruiting
Cyclophosphamide/Fludarabine	NY-ESO-1-157-165 peptide pulsed dendritic cell vaccine	Malignant neoplasm	II	6	NCT01697527	Active, not recruiting
	Autologous dendritic cells loaded with autologous tumor-lysate	Melanoma	I	20	NCT01946373	Recruiting
	MART-1 peptide-pulsed dendritic cells	Melanoma	II	1230	NCT00338377	Active, not recruiting
Cyclophosphamide	Autologous dendritic cell vaccine loaded with personalized peptides	Non-small cell lung cancer	I	16	NCT05195619	Recruiting
Gemcitabine	Autologous DC vaccine	Sarcoma	I	19	NCT01803152	Active, not recruiting
Platinum/Pemetrexed	Dendritic cells loaded with the mesothelioma-associated tumor antigen Wilms’ tumor protein 1	Malignant pleural mesothelioma	I/II	28	NCT02649829	Active, not recruiting
Decitabine	Dendritic cell/acute myelogenous leukemia fusion cell vaccine	Acute myelogenous leukemia	I	45	NCT03679650	Recruiting

**Table 3:** Active clinical trials combining DC-based vaccine with targeted drugs (clinicaltrials.gov, May 28, 2023).

Intervention		Tumor	Phase	*N*	Trial identifier	Status
Targeted drug(s)	DC-based vaccine used					
Trastuzumab/Pepinemab	Dendritic cell (DC1) vaccine	Breast cancer	I	28	NCT05378464	Recruiting
Trastuzumab/Pertuzumab	HER-2 pulsed DC1	Breast cancer	II	53	NCT05325632	Recruiting

## 5 Conclusion

DCs play an indispensable role in triggering anti-tumor immune responses. However, under tumor conditions, immunosuppressive TME weakens their function. The defective function of DCs is an important reason why tumors evade immune surveillance and is closely associated with the poor efficacy of some immunotherapies, such as immune checkpoint inhibitors. Based on the pivotal role of DCs in the immune response, which determines their importance in anti-tumor therapy, many studies have been undertaken to improve the function of DCs, and some protocols, such as DC-based vaccines, have become available options for the treatment of tumors. In addition, the use of DC-based vaccines in combination with ICIs has good application prospects because they can induce a more effective baseline immune response, which is necessary for ICIs to exert their anticancer effects. However, several issues remain unaddressed. The complex composition of the TME and the close and diverse interactions among its components ultimately result in the inhibition of the normal function of multiple immunostimulatory cells, including DCs, and the induction of immune escape. How to effectively and selectively target the immunosuppressive effects of the TME on DCs needs to be further explored.

## Author contributions

ZZ designed the study. ZX and RW wrote the manuscript and generated the figures. XW and HY revised and reviewed the manuscript. JD, XH, YY, JG, and JC contributed to the conceptualization and critically edited the manuscript. All authors contributed to the article and approved the submitted version.
